# Supplementary figures and images for: Immune-related hub genes in intrauterine adhesions: a bioinformatics approach
Source: PeerJ. 2025 Oct 3;13:e20035. doi: 10.7717/peerj.20035 (PMC12499562; doi:10.7717/peerj.20035)

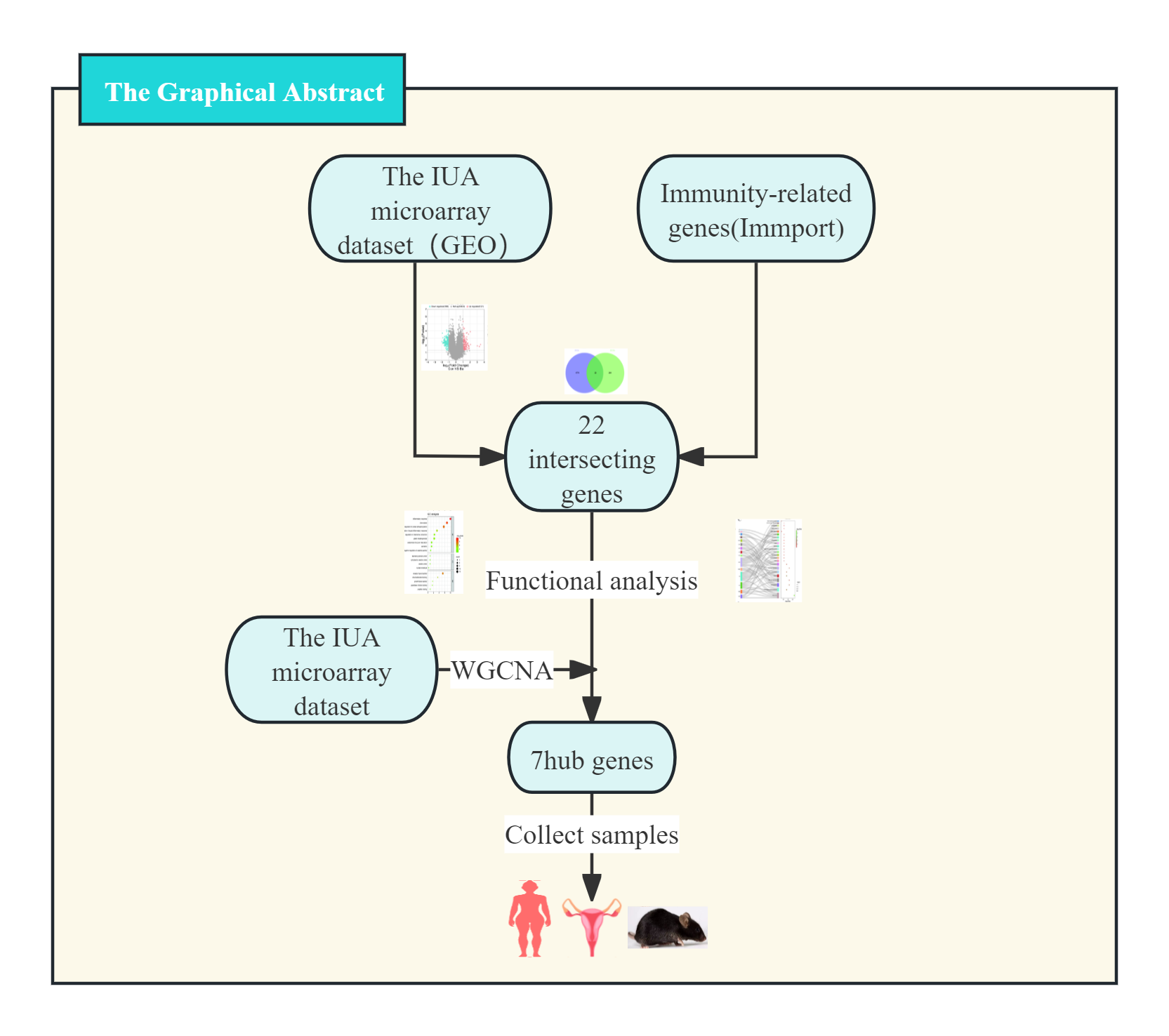

Supplement: Supplemental Information 6 [file peerj-13-20035-s006.png]
