# Supplementary material for: Immune-related hub genes in intrauterine adhesions: a bioinformatics approach
Source: PeerJ. 2025 Oct 3;13:e20035. doi: 10.7717/peerj.20035 (PMC12499562; doi:10.7717/peerj.20035)
Supplement: Supplemental Information 7 [file peerj-13-20035-s007.doc]

微生信示例 t011输出 The Weiboshengxin example t011 output
